# NEAT1 Promotes LPS-induced Inflammatory Injury in Macrophages by Regulating MiR-17-5p/TLR4

**DOI:** 10.1515/med-2020-0007

**Published:** 2020-01-17

**Authors:** Yanhui Li, Wei Guo, Yeping Cai

**Affiliations:** 1 ChenZhou NO.1 People's Hospital LuoJiaJin, ChenZhou China; 2 ICU 1 Zone, ChenZhou NO.1 People's Hospital, ChenZhou, HuNan, 423000, China

**Keywords:** Sepsis, Inflammatory response, NEAT1, miR-17-5p, TLR4

## Abstract

**Background:**

The inflammatory response of macrophages is responsible for sepsis. Long noncoding RNA nuclear enriched abundant transcript 1 (NEAT1) has been reported to be involved in sepsis development. However, its underlying mechanism remains largely unclear. This study aims to investigate the effect of NEAT1 on inflammatory response of macrophages and explore the regulatory network of NEAT1/microRNA-17-5p (miR-17-5p)/Toll-like receptor 4 (TLR4).

**Methods:**

The serum samples of 68 sepsis patients and 32 heathy controls were collected. THP-1 macrophages were treated with lipopolysaccharide (LPS) to induce inflammatory injury model of sepsis. The expressions of NEAT1, miR-17-5p and TLR4 were measured by quantitative real-time polymerase chain reaction or western blot. The inflammatory response was investigated by levels of inflammatory cytokines, tumor necrosis factor-alpha (TNF-ɑ), interleukin-1beta (IL-1β) and IL-6 as well as nitric oxide (NO) production. The interaction among NEAT1, miR-17-5p and TLR4 were investigated by bioinformatics analysis, luciferase reporter assay and RNA pull-down.

**Results:**

NEAT1 expression was enhanced in patient serum and associated with severity of sepsis. Knockdown of NEAT1 inhibited levels of TNF-ɑ, IL-1β, IL-6 and NO release in LPS-treated macrophages. miR-17-5p is bound to NEAT1 and its abrogation reversed NEAT1 knockdown-mediated inhibition of inflammatory response in LPS-treated macrophages. Overexpression of miR-17-5p weakened LPS-induced inflammatory response. TLR4 as a target of miR-17-5p was regulated by NEAT1 and miR-17-5p. TLR4 res-to ration alleviated silencing NEAT1-induced inflammatory suppression.

**Conclusion:**

Silence of NEAT1 suppressed LPS-induced inflammatory response of macrophages by mediating miR-17-5p and TLR4, indicating that NEAT1 might be a promising target for sepsis treatment.

## 1 Introduction

Sepsis is a complex inflammatory syndrome in response to infection with high mortality, possibly leading to organ dysfunction [1]. Inflammatory response is a key pathogenesis of sepsis [2]. Macrophage development and function play important roles in inflammatory disorders, including sepsis [3]. However, effective strategies for sepsis treatment are limited in clinic.

Noncoding RNAs, including long noncoding RNAs (lncRNAs), microRNAs (miRNAs) and circular RNA, have been reported to be associated with sepsis development by regulating innate immunity or other biological processes [4]. Therein, lncRNAs are dysregulated and involved in inflammatory response as well as immunopathology of sepsis [5]. LncRNA nuclear enriched abundant transcript 1 (NEAT1), an attractive biomarker in human cancers and diseases, has been suggested to facilitate inflammatory response [6, 7]. Moreover, emerging evidence suggests that in sepsis patients, NEAT1 is correlated with increasing risk, severity and poor prognosis [8]. However, little is known about the mechanism which allows NEAT1 participation in sepsis development.

miRNAs also serve as regulator of inflammatory response and have essential roles in diagnosis and treatment of sepsis [9]. miR-17-5p has been shown to have an impact on the outcome of various disorders [10, 11, 12]. More particularly, miR-17-5p has been suggested as an inflammation-related miRNA and might be associated with sepsis-induced acute lung injury [13, 14]. Toll-like receptor 4 (TLR4) activated by bacterial endotoxin-like lipopolysaccharide (LPS) is required for initiating inflammatory response and responsible for sepsis [15]. Anti-TLR4 therapy has been regarded as promising avenue for sepsis [16]. Intriguingly, Ji et al. reported that Schisandrin B could inhibit inflammatory response in LPS-induced sepsis by regulating miR-17-5p and TLR4 [17]. Based on the bioinformatics prediction, NEAT1 might target miR-17-5p. Hence, we hypothesized that miR-17-5p and TLR4 might be involved in NEAT1-mediated inflammation in sepsis. In this study, we measured the expression of NEAT1 in sepsis patients. Moreover, we investigated the effect of NEAT1 on inflammatory response in LPS-treated macrophages and explored the potential interaction of NEAT1/miR-17-5p/ TLR4.

## 2 Materials and methods

### 2.1 Sepsis patients and sample collection

This study was approved by the Ethics Committee of Chen-Zhou NO.1 People’s Hospital and conducted in accordance with the Declaration of Helsinki. A total of 68 sepsis patients and 32 healthy controls with written informed consent were recruited. The basic patient characteristics are shown in Table 1. Blood samples were collected from participants and centrifuged at 3000 rpm for 20 min. Serum (supernatant) was maintained at -80°C until used.

**Table 1:** Clinical characteristics of the patients in this study.

		Sepsis patients (N=68)			
characteristics	Healthy control (n=32)	Sepsis subgroup (n=23)	Severe sepsis subgroup (n=30)	Septic shock subgroup (n=15)	P-Value
Age	42.3±13.9	45.8±15.2	47.2±16.1	46.5±14.3	0.597
Sex					
Male	17	12	13	8	0.857
Female	15	11	17	7	
Blood culture					
Negative	NA	4	5	2	0.872
Bacterial, Gram-positive	NA	5	8	2	
Bacterial, Gram-negative	NA	12	16	9	
Fungi	NA	2	1	2	
Primary infection site					
Respiratory tract	NA	7	12	7	0.896
Urinary tract	NA	6	7	3	
Abdomen	NA	10	11	5	
SOFA score (range)	NA	5.6±2.5	8.0±2.1	10.0±1.0	<0.001
C-reactive protein, mg/l (range)	NA	52.5±27.4	68.3±24.1	82.4±25.4	0.003
ICU stay, days, median (range)	NA	13±4	17.5±3	23±3	<0.001
28-day survival					
Yes	NA	16	21	4	0.011
No	NA	7	9	11	

NA, not applicable; SOFA, Sepsis-related Organ Failure Assessment; ICU, intensive care unit

### 2.2 Cell culture and treatment

THP-1 macrophages were cultured in RPMI1640 medium (Sigma, St. Louis, MO, USA) supplemented with 10% fetal bovine serum and penicillin/streptomycin at 37°C and 5% CO_2_. To establish inflammatory injury model, cells were exposed with 1 μg/mL LPS (Sigma, St. Louis, MO, USA) for 24 h before harvest.

Small interfering RNA (siRNA) targeting NEAT1 (siNEAT1), siRNA negative control (scrambled), pcD-NA-NEAT1 overexpression vector (NEAT1), TLR4 overexpression vector (TLR4), pcDNA vector, miR-17-5p mimic (miR-17-5p), miRNA negative control (miR-NC), miR-17-5p inhibitor (anti-miR-17-5p) and inhibitor negative control (anti-miR-NC) were synthesized by Genepharma (Shanghai, China). The macrophages were seeded at a density of 0.5 × 10^6^ cells/ml for 24 h and then transfected with the oligonucleotides (50 nM) or vectors using Lipofectamine 3000 transfection reagent (Invitrogen, Carlsbad, CA, USA). After 24 h of transfection, cells were collected for the following analyses.

### 2.3 Quantitative real-time polymerase chain reaction (qRT-PCR)

Serum RNA was extracted using ISOLATE II Biofluids RNA Kit (Bioline, London, UK) according to the manufacturer’s instructions. Cell RNA was extracted using Trizol reagent (Invitrogen, Carlsbad, CA, USA). The RNA was used for cDNA synthesis and qRT-PCR via All-in-One^TM^ qRT-PCR Detection Kit (GeneCopoeia, Rockville, MD, USA). With U6 small RNA or GAPDH as endogenous references, the relative expressions of miR-17-5p, NEAT1, TNF-α, IL-1β, IL-6 and TLR4 were analyzed via 2^-ΔΔCt^ method [18]. The primers for miR-17-5p or U6 were provided by GeneCopoeia and primers for NEAT1, tumor necrosis factor-alpha (TNF-ɑ), interleukin-1beta (IL-1β), IL-6, TLR4 or GAPDH were obtained from Sangon Biotech (Shanghai, China): NEAT1 (Forward, 5’-CAGTTAGTTTAT-CAGTTCTCCCATCCA-3’; Reverse, 5’-GTTGTTGTCGTCACCT-TTCAACTCT-3’), TNF-α (Forward, 5’-TTACGCCTTTGAA-GTTAGCAG-3’; Reverse, 5’-CGTCCAAATACATCGCAAC-3’), IL-6 (Forward, 5’-TACTCGGCAAACCTAGTGCG-3’; Reverse, 5’-GTGTCCCAACATTCATATTGTCAGT-3’), IL-1β (Forward, 5’-TCTTTGAAGAAGAGCCCGTCCTC-3’; Reverse, 5’-GGATCCACACTCTCCAGCTGCA-3’), TLR4 (Forward, 5’-GGTGATTGTTGTGGTGTCCCA-3’; Reverse, 5’-AGTGTTCCTGCTGAGAAGGCG-3’), GAPDH (Forward, 5’-GATATTGTTGCCATCAATGAC-3’; Reverse, 5’-TTCTCCAT-GGTGGTGAAGACGCCA-3’).

### 2.4 Nitric oxide (NO) release and inflammatory cytokine secretion analyses

After treatment of LPS for 24 h, the supernatant of macrophages medium was collected. NO release was determined by NO Colorimetric Assay Kit (Beyotime, Shanghai, China) and the absorbance at 540 nm was measured with a microplate reader (Bio-Rad, Hercules, CA, USA). Levels of TNF-α, IL-1β and IL-6 in medium were detected by special commercial enzyme-linked immunosorbent assay (ELISA) kits (Sigma, St. Louis, MO, USA) according to the manufacturer’s instructions. The abundance of inflammatory cytokine was calculated by the absorbance at 450 nm and a standard curve.

### 2.5 Bioinformatics analysis and luciferase reporter assay

Bioinformatics analysis provided the potential binding sites of miR-17-5p and NEAT1 or TLR4 by starBase v2.0 and TargetScan Release 7.2. NEAT1 contains two seed sites of miR-17-5p (chr11: 65206890-65206912 and 65210090-65210111). TLR4 includes two binding sites of miR-17-5p at its 3’ untranslated regions (3’-UTR) (position 7813-7819 and 9155-9161). The wild type (Wt) or mutant (Mut) luciferase reporter construct for NEAT1 or TLR4 was generated using pGL3 vector (Promega, Madison, WI, USA), named as NEAT1-Wt, NEAT1-Mut1, NEAT1-Mut2, NEAT1-Mut1&2, TLR4-Wt, TLR4-Mut1, TLR4-Mut2 or TLR4-Mut1&2, respectively. For luciferase reporter assay, macrophages were seeded in 24-well plates in a density of 3 × 10^4^ cells/well overnight and then co-transfected with Wt or Mut luciferase reporter vector and miR-17-5p, miR-NC, anti-miR-17-5p or anti-miR-NC using Lipofectamine 3000 transfection reagent. A dual luciferase reporter assay kit (Promega, Madison, WI, USA) was used for luciferase activity assay in each group after 48 h of post-transfection.

### 2.6 RNA pull-down

The Wt or Mut sequences of miR-17-5p containing the potential binding sites of NEAT1 were synthesized and labeled with biotin to be Bio-miR-17-5p-Wt or Bio- miR-17-5p-Mut. Bio-miR-NC was used as the control. Macrophages were lysed in RIPA buffer (Beyotime, Shanghai, China) with RNase inhibitor (Invitrogen, Carlsbad, CA, USA) and incubated with Bio-miR-17-5p-Wt, Bio-miR-17-5p-Mut or Bio-miR-NC for 2 h at 4°C, followed by interacting with streptavidin beads for 1h. The bound RNAs were extracted and NEAT1 enrichment in pull-down products was detected via qRT-PCR analysis.

### 2.7 Western blot

Macrophages were washed and lysed in RIPA buffer, followed by centrifuging at 12,000 rpm for 10 min. Total proteins (20 μg/well) in supernatant were separated by 10% SDS-PAGE and transferred to polyvinylidene difluoride membranes (Millipore, Billerica, MA, USA). After blocking the non-specific sites, membranes were incubated with rabbit polyclonal to TLR4 or GAPDH (Abcam, Cambridge, MA, USA) overnight at 4°C, and then with horseradish peroxidase-conjugated anti-rabbit secondary antibody for 2 h. The blot signaling was detected using enhanced chemiluminescence chromogenic substrate (Beyotime, Shanghai, China) and X-OMAT BT film (Carestream Health, Rochester, NY, USA). The quantitative assay was performed using Quantity One software (Bio-Rad, Hercules, CA, USA) and expressed as ratio of TLR4/GAPDH levels.

### 2.8 Statistical analysis

GraphPad Prism 7 (GraphPad Inc., La Jolla, CA, USA) was used for statistical analysis based on three independent experiments. The data were presented as the mean ± standard deviation (S.D.). Spearman’s correlation analysis was conducted to analyze the association between NEAT1 expression and Sepsis-related Organ Failure Assessment (SOFA) score in sepsis patients. Student’s *t* test was used for comparisons between two groups and one-way analysis of variance (ANOVA) was used for multiple groups comparisons. *P*<0.05 was regarded as significant.

**Ethical statement**: This study was approved by the Ethics Committee of ChenZhou NO.1 People’s Hospital and conducted in accordance with the Declaration of Helsinki.

## 3 Results

### 3.1 NEAT1 is highly expressed in sepsis patients

**Figure 1:**
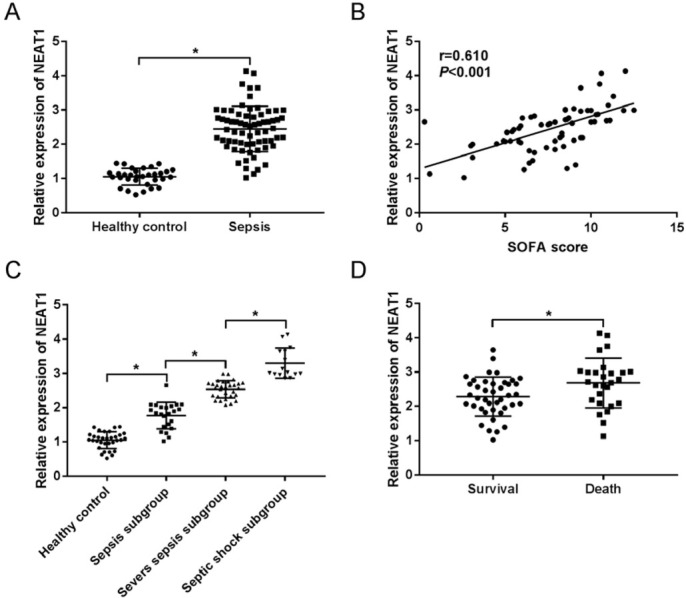
The expression of NEAT1 is increased in sepsis patients. (A) The expression of NEAT1 was measured in healthy control and sepsis samples by qRT-PCR. (B) The association between NEAT1 expression and patient SOFA score. (C) The level of NEAT1 was measured in healthy control and in patients with different severities of sepsis. (D) The abundance of NEAT1 was measured in survival or death group. *P<0.05.

To explore the potential role of NEAT1 in sepsis development, its expression was measured in serum of sepsis patients. Compared with healthy control, sepsis group displayed abnormally elevated NEAT1 level (Figure 1A). Moreover, the abundance of NEAT1 was positively correlated with SOFA score of patients (Figure 1B). As shown in Figure 1C, NEAT1 expression was progressively enhanced with the increasing severity of patients. Meanwhile, patients were classified as death or survival group after a 28-day follow up. The assay of qRT-PCR revealed that death group showed higher NEAT1 level than survival group (Figure 1D). These data suggested that high expression of NEAT1 might be associated with poor outcome of sepsis patients.

### 3.2 NEAT1 knockdown inhibits LPS-induced inflammatory response in macrophages

**Figure 2:**
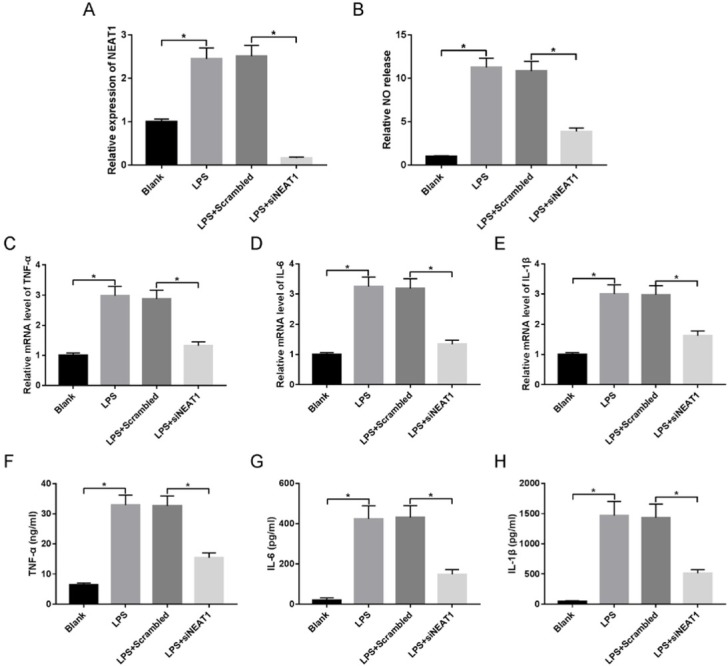
NEAT1 silence inhibits LPS-induced inflammatory injury in macrophages. Macrophages were transfected with siNEAT1 or scrambled and then treated with LPS. NEAT1 expression (A), NO release (B), and inflammatory cytokines secretion (C-H) in each group were measured by qRT-PCR, NO kit or ELISA kit. *P<0.05.

To investigate the effect of NEAT1 on inflammatory injury, macrophages were transfected with siNEAT1 or scrambled and then treated with LPS for 24h. The results of qRT-PCR showed that NEAT1 expression in macrophages was significantly enhanced by LPS treatment compared with that in blank group, while it was effectively decreased by transfection of siNEAT1 (Figure 2A). Subsequently, inflammatory response was investigated by NO production and inflammatory expression. As demonstrated in Figure 2B, compared with that in non-treated cells, NO release was obviously enhanced in macrophages after LPS stimulation, while it was strongly suppressed by down-regulation of NEAT1. Meanwhile, treatment with LPS induced remarkable inflammatory response in macrophages resulting in increase of TNF-ɑ, IL-1β and IL-6 at mRNA and protein levels (Figure 2C-2H). However, silencing NEAT1 played an opposite effect on their expressions.

### 3.3 Abrogation of miR-17-5p reverses silencing NEAT1-mediated inhibition of LPS-induced inflammation response in macrophages

**Figure 3:**
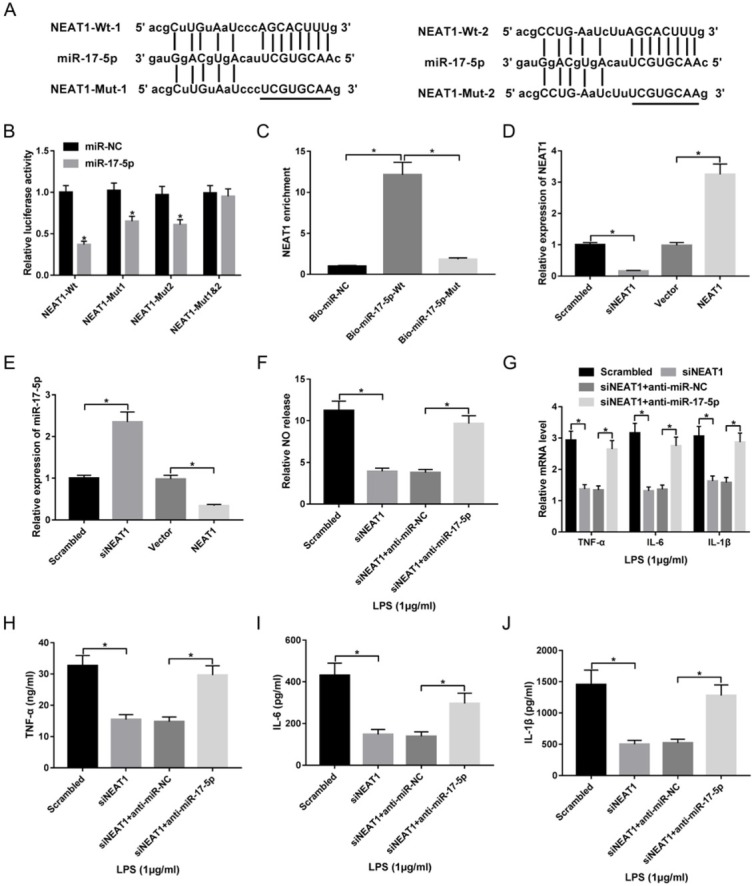
NEAT1 knockdown suppresses LPS-induced inflammatory injury by targeting miR-17-5p in macrophages. (A) The potential binding sites of NEAT1 and miR-17-5p. (B) Luciferase activity was detected in macrophages co-transfected with miR-17-5p or miR-NC and NEAT1-Wt or NEAT1-Mut. (C) The enrichment of NEAT1 was detected in macrophages by RNA pull-down. (D and E) The expressions of NEAT1 and miR-17-5p were detected in macrophages transfected with scrambled, siNEAT1, vector or NEAT1. (F-J) NO release and inflammatory cytokines levels were measured in macrophages transfected with scrambled, siNEAT1, siNEAT1 and anti-miR-NC or anti-miR-17-5p. *P<0.05.

To explore how NEAT1 participates in inflammatory response, bioinformatics analysis was performed to explore its potential target by starBase v2.0, describing two promising binding sites with miR-17-5p, suggesting that miR-17-5p might be bound to NEAT1 (Figure 3A). To validate this prediction, NEAT1-Wt or NEAT1-Mut luciferase reporter vector was made and co-transfected with miR-17-5p or miR-NC into macrophages. Results showed that overexpression of miR-17-5p significantly decreased the luciferase activity, while its efficacy was weakened by mutating the putative binding region 1 or 2 and even lost by combined mutant (Figure 3B). Meanwhile, the data by biotin RNA pull down, as shown in Figure 3C, show that biotinylated miR-17-5p led to obvious increase of NEAT1 enrichment, whereas it was abolished by mutating the binding sites of miR-17-5p and NEAT1. Moreover, the effect of NEAT1 on miR-17-5p expression was measured in macrophages by overexpressing or silencing NEAT1. The transfection efficacy was validated in Figure 3D and miR-17-5p expression level was negatively regulated by NEAT1 (Figure 3E). To explore whether miR-17-5p is involved in NEAT1-mediated regulation of inflammatory response, macrophages were transfected with scrambled, siNEAT1, siNEAT1 and anti-miR-NC or anti-miR-17-5p and then treated with LPS. As displayed in Figure 3F-3J, deficiency of miR-17-5p abated the suppressive impact of NEAT1 silence on NO release and inflammatory cytokines expressions in LPS-treated macrophages.

### 3.4 Overexpression of miR-17-5p suppresses LPS-induced inflammatory response in macrophages

**Figure 4:**
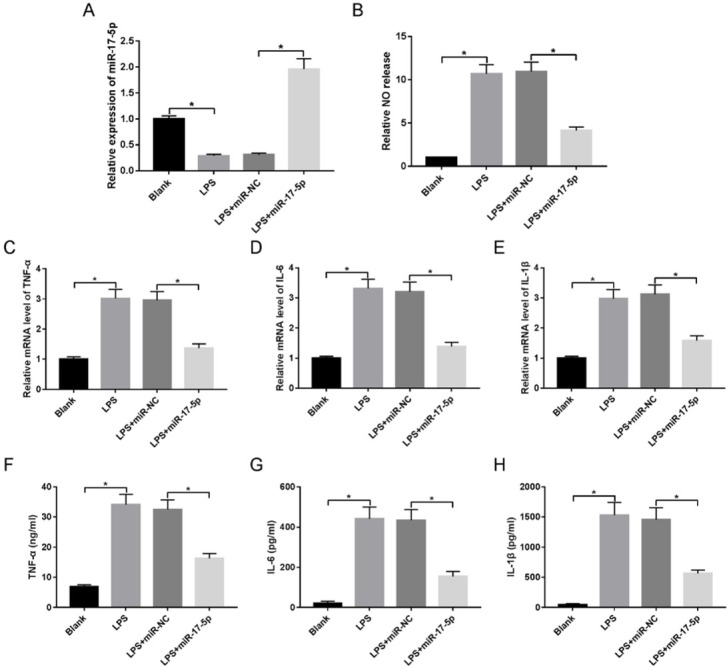
miR-17-5p overexpression suppresses LPS-induced inflammatory injury in macrophages. Macrophages were transfected with miR-NC or miR-17-5p and then treated with LPS. miR-17-5p expression (A), NO release (C) and inflammatory cytokine expressions (C-H) were measured in transfected cells. *P<0.05.

To evaluate the biological role of miR-17-5p in inflammatory response, macrophages were transfected with miR-17-5p or miR-NC followed by treatment of LPS. As a result, the abundance of miR-17-5p was notably reduced in macrophages after exposure to LPS, while it was restored by transfection of miR-17-5p (Figure 4A). The assay of NO production showed that up-regulation of miR-17-5p greatly inhibited LPS-induced NO release in macrophages (Figure 4B). Furthermore, the levels of TNF-ɑ, IL-1β and IL-6 mRNA in cells and protein in medium induced with LPS were evidently impeded via overexpressing miR-17-5p (Figure 4C-4H).

### 3.5 TLR4 is regulated by NEAT1 and miR-17-5p

**Figure 5:**
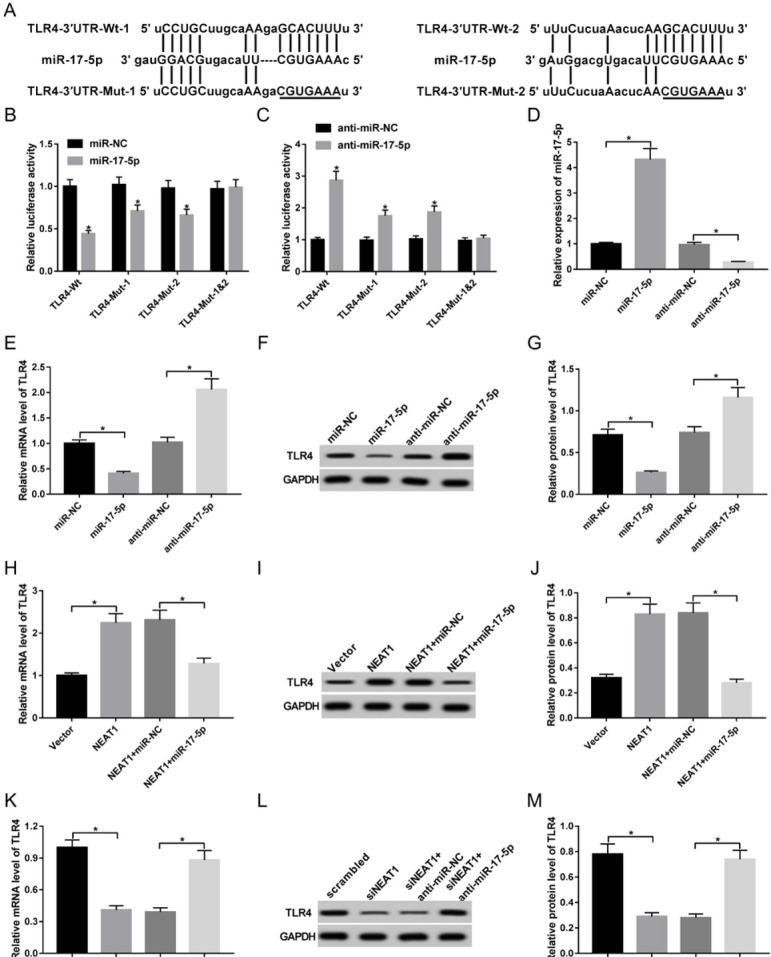
TLR4 is regulated by NEAT1 and miR-17-5p in macrophages. (A) The potential binding sites of miR-17-5p and TLR4. (B and C) Luciferase activity was detected in macrophages co-transfected with miR-17-5p, miR-NC, anti-miR-17-5p or anti-miR-NC and TLR4-Wt or TLR4-Mut. (D) The expression of miR-17-5p was detected in macrophages transfected with miR-NC, miR-17-5p, anti-miR-NC or anti-miR-17-5p. (E-G) The expression of TLR4 mRNA and protein was measured in macrophages transfected with miR-NC, miR-17-5p, anti-miR-NC or anti-miR-17-5p. (H-M) The level of TLR4 mRNA and protein was detected in macrophages transfected with vector, NEAT1, NEAT1 and miR-NC or miR-17-5p, scrambled, siNEAT1, siNEAT1 and anti-miR-NC or anti-miR-17-5p. *P<0.05.

To further explore the mechanism of NEAT1 in inflammatory response, target of miR-17-5p was predicted by bio-informatics analysis using TargetScan Release 7.2. TLR4 was suggested as a target of miR-17-5p with two potential binding positions (Figure 5A). Luciferase reporter assay was conducted to identify their association with the results that overexpression of miR-17-5p conspicuously inhibited the luciferase activity of TLR4-Wt group, while miR-17-5p played an opposite effect, which were counteracted by mutating the binding sites (Figure 5B and 5C). Moreover, the effect of miR-17-5p on TLR4 expression was investigated in macrophages transfected with miR-NC, miR-17-5p, anti-miR-NC or anti-miR-17-5p. Transfection efficacy was revealed by obvious up-regulation or down-regulation of miR-17-5p level (Figure 5D). Meanwhile, the expression of TLR4 was significantly decreased by miR-17-5p overexpression and enhanced by miR-17-5p knockdown at mRNA and protein levels (Figure 5E-5G). In addition, the level of TLR4 was measured in cells transfected with vector, NEAT1, NEAT1 and miR-NC or miR-17-5p. Results demonstrated that overexpression of NEAT1 induced increased expression of NEAT1 mRNA and protein, which was attenuated by introduction of miR-17-5p (Figure 5H-5J). However, the opposite occurred by down-regulating NEAT1 or along with miR-17-5p (Figure 5K-5M).

### 3.6 TLR4 up-regulation ameliorates silence of NEAT1-mediated suppression of LPS-induced inflammatory response in macrophages

**Figure 6:**
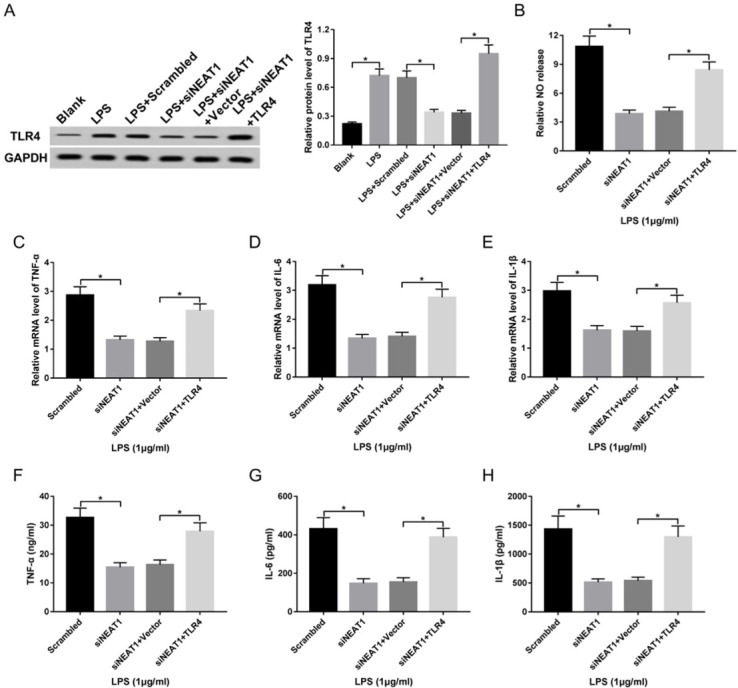
TLR4 reverses silence of NEAT1-mediated inhibition of LPS-induced injury in macrophages. Cells were transfected with scrambled, siNEAT1, siNEAT1 and vector or TLR4 and then treated with LPS. TLR4 protein level (A), NO release (B), and inflammatory cytokine expression (C-H) were measured in the cells. *P<0.05.

To evaluate whether NEAT1-addressing inflammatory response was modulated by TLR4, macrophages were transfected with scrambled, siNEAT1, siNEAT1 and TLR4 or vector and treated with LPS. As shown in Figure 6A, TLR4 protein level was significantly enhanced in macrophages by LPS treatment, while it was decreased by knockdown of NEAT1, which was restored by introduction of TLR4 overexpression vector. Besides, restoration of TLR4 significantly mitigated interference of NEAT1-induced inhibitive role in NO production and TNF-ɑ, IL-1β and IL-6 expression at mRNA and protein levels in LPS-challenged macrophages (Figure 6B-6H).

## 4 Discussion

Noncoding RNAs have been regarded as potential targets for therapeutics of sepsis [19]. Previous work showed that NEAT1 was highly expressed in sepsis-induced acute kidney injury patients and aggravated LPS-induced injury [20]. This report shows that NEAT1 might play an important role in sepsis. In our study, NEAT1 expression was enhanced in sepsis patients and was associated with severity of patients; suggesting that NEAT1 might be a promoter of sepsis development, as previously shown [8]. However, how NEAT1 mediates sepsis development is largely unknown. Here we first provided the regulatory network of NEAT1/miR-17-5p/TLR4, revealing novel theoretical basis for application of NEAT1 in sepsis treatment.

The pathogenesis of sepsis is characterized by increased inflammatory response, such as cytokine secretion. NO is produced by nitric oxide synthase, which is mediated by cytokines or LPS [21]. Therefore, release of NO is an important monitor for inflammatory response in macrophages. In accordance with these mentioned views, the inflammatory injury model was established by LPS-treated macrophages. We found that treatment with LPS successfully triggered inflammatory response, revealed by increase of NO release and pro-inflammatory cytokine secretion. Thereby down-regulating NEAT1 through siRNA attenuated LPS-induced inflammatory response in macrophages, indicating the potential therapeutic effect of NEAT1 in sepsis.

Evidence in support of the interaction between lncRNA and miRNA indicates the main mechanism of competing endogenous RNA (ceRNA) regulatory network [22]. Recent finding summarized that NEAT1 could also serve as a ceRNA, which regulates sepsis-induced acute kidney injury by sponging miR-204 to target IL-6R and mediating nuclear factor kappa B (NF-κB) pathway [20]. Furthermore, NEAT1 was reported as a decoy of miR-107 in gastric cancer cells [23]. As a mature miRNA of miR-17, miR-17-5p was validated in this study to be bound and negatively regulated by NEAT1 in macrophages.. Based on NEAT1 suppression, miR-17-5p deficiency can reverse the effect of NEAT1 silence on LPS-induced inflammatory response in macrophages, indicating that the effect of NEAT1 on LPS-induced inflammatory response was achieved by sponging miR-17-5p. To further elucidate the ceRNA network, considering that functional miRNA is known to regulate its target gene expression, the potential target of miR-17-5p was explored by bioinformatics analysis. Previous studies have revealed transforming growth factor-β receptor 2, SRC kinase signaling inhibitor 1, phosphatase and tensin homologue and cyclin-dependent kinase inhibitor 1 as functional targets of miR-17-5p in multiple conditions [10, 11, 12]. In this study, as indicated by luciferase reporter assay, we identified TLR4 as a target of miR-17-5p, which is also consistent with a previous report [17]. Besides, TLR4 expression positively correlated with NEAT1 abundance, which was attenuated by miR-17-5p, suggesting that NEAT1 could derepress TLR4 by sponging miR-17-5p.

TLR4 as the main receptor of LPS is activated in LPS-challenged macrophages which in turn induced immune cells to release inflammatory cytokines, such as TNF-ɑ, IL-1β and IL-6, producing systemic inflammatory response. Previous efforts suggest that TNF-ɑ was increased in many inflammatory processes and predicted an increased risk of sepsis after burn injury [24, 25, 26]. Moreover, it was demonstrated that TLR4 exhaustion contributed to resistance in immune dysfunction induced by sepsis [27]. TLR4 interference have been regarded as a potential therapeutic target for sepsis as well as sepsis-induced organ injury [28, 29]. In this study, TLR4 restoration reversed the suppressive effect of NEAT1 knockdown on LPS-induced inflammatory response, uncovering TLR4 as a therapeutic target of NEAT1, which is consistent with a previous report [30]. Previous study suggest that LPS-initiated TLR4 signaling mediates mitogen activated protein kinases (MAPK) and NF-κB pathways [31]. Hence, these downstream signaling of TLR4 should be further explored. Moreover, to better elucidate the mechanism, an animal model of sepsis is required for further studies.

## 5 Conclusion

The results of this study show that high expression of NEAT1 is associated with severity of sepsis and knockdown of NEAT1 attenuates LPS-induced inflammatory response in macrophages, possibly by acting as a ceRNA of miR-17-5p to regulate TLR4. Our study indicates that NEAT1 might serve as a promising target for diagnosis and therapeutics of sepsis.
